# The Effect of Hexamethylene Diisocyanate-Modified Graphene Oxide as a Nanofiller Material on the Properties of Conductive Polyaniline

**DOI:** 10.3390/polym11061032

**Published:** 2019-06-11

**Authors:** José Antonio Luceño Sánchez, Ana Maria Díez-Pascual, Rafael Peña Capilla, Pilar García Díaz

**Affiliations:** 1 Department of Analytical Chemistry, Physical Chemistry and Chemical Engineering, Faculty of Sciences, Alcalá University, 28805 Madrid, Spain; jose.luceno@uah.es; 2 Department of Signal Theory and Communication, Polytechnic High School, Alcalá University, 28805 Madrid, Spain; rafa.pena@uah.es (R.P.C.); pilar.garcia@uah.es (P.G.D.)

**Keywords:** PANI, graphene oxide, hexamethylene diisocyanate, nanocomposite, thermal stability, mechanical properties

## Abstract

Conducting polymers like polyaniline (PANI) have gained a lot of interest due to their outstanding electrical and optoelectronic properties combined with their low cost and easy synthesis. To further exploit the performance of PANI, carbon-based nanomaterials like graphene, graphene oxide (GO) and their derivatives can be incorporated in a PANI matrix. In this study, hexamethylene diisocyanate-modified GO (HDI-GO) nanosheets with two different functionalization degrees have been used as nanofillers to develop high-performance PANI/HDI-GO nanocomposites via in situ polymerization of aniline in the presence of HDI-GO followed by ultrasonication and solution casting. The influence of the HDI-GO concentration and functionalization degree on the nanocomposite properties has been examined by scanning electron microscopy (SEM), Raman spectroscopy, X-ray diffraction (XRD), thermogravimetric analysis (TGA), tensile tests, zeta potential and four-point probe measurements. SEM analysis demonstrated a homogenous dispersion of the HDI-GO nanosheets that were coated by the matrix particles during the in situ polymerization. Raman spectra revealed the existence of very strong PANI-HDI-GO interactions via π-π stacking, H-bonding, and hydrophobic and electrostatic charge-transfer complexes. A steady enhancement in thermal stability and electrical conductivity was found with increasing nanofiller concentration, the improvements being higher with increasing HDI-GO functionalization level. The nanocomposites showed a very good combination of rigidity, strength, ductility and toughness, and the best equilibrium of properties was attained at 5 wt % HDI-GO. The method developed herein opens up a versatile route to prepare multifunctional graphene-based nanocomposites with conductive polymers for a broad range of applications including flexible electronics and organic solar cells.

## 1. Introduction

Over the last years, conducting polymers such as polypyrrol, polythiophene and polyaniline (PANI) have attracted a lot of interest both at an academic and industrial level owing to their exceptional electrical and optoelectronic properties arising from their expanded π-conjugated electron system, which make them suitable for a wide range of applications, including thermoelectric devices, batteries, solar cells, sensors, actuators, supercapacitors, memory devices, wastewater treatment, separation ions and so forth [1,2,3]. Amongst them, PANI, a semi-flexible rod polymer, has been one of the most investigated over the last 20 years due to its unique electrochemical performance, adjustable electrical conductivity, facile synthesis, good chemical, environmental and thermal stability, easy doping chemistry, inexpensiveness and numerous uses [1,4]. It can be prepared with a controllable conductivity by adjusting the pH using both the conventional chemical and electrochemical approaches. 

**Scheme 1:**
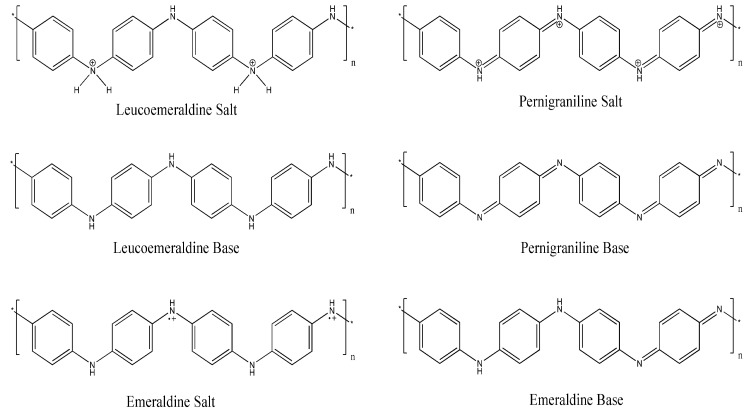
Structure of the different forms of polyaniline (PANI): The completely reduced leucoemeraldine, the completely oxidized pernigraniline and the emeraldine, in the base and salt forms.

PANI can be polymerized into three different oxidation states [5]: The completely reduced leucoemeraldine form, the fully oxidized pernigraniline form and the emeraldine form, comprising oxidized and reduced alternating repeating units (Scheme 1). Further, each form may occur as either a salt or a base, and they have different colours, stabilities, and conductivities. Leucoemeraldine is a colourless substance that slowly oxidizes in air and is electrically insulating. It can be oxidized in an acidic medium (p-doping) to yield the conducting emeraldine salt. Pernigraniline is a blue insulating compound unstable in water that easily decomposes in air. The green emeraldine salt is formed during protonation of the violet emeraldine base with organic and inorganic acids: The protons interact with the imine nitrogen atoms leading to the formation of polycations [6]. The positive charges onto neighbouring nitrogen atoms rise the energy of the polymeric system, hence electron density undergoes redistribution; as a consequence, “unpairing” of the electron pair of nitrogen atoms occurs and cation radicals appear; these are delocalized over a certain conjugation length and provide the electron conductivity of the polymer, which depends strongly on the degree of protonation [5].

Despite the wide spectrum of properties of PANI, it presents several shortcomings for certain applications, namely poor solubility, low mechanical performance and short cycling life [7]. To overcome these issues, PANI can be polymerized in the presence of organic and inorganic nanomaterials. The tailored incorporation of nanoscale materials can lead to nanocomposites with improved properties due to synergistic effects [8]. Thus, various carbon-based materials like mesoporous carbon, carbon nanotubes and graphene have been used as additives to extend the functional uses and improve the performance of PANI. In particular, composites of PANI reinforced with graphite, graphene (G), graphene oxide (GO) and reduced graphene oxide (rGO) have been synthesized via in situ chemical or electrochemical polymerization, covalent and non-covalent functionalization as well as self-assembly strategies [8,9,10,11,12]. The nanomaterial is supposed to increase the specific surface area of the composites, reduce the mechanical deformation of PANI and improve the transportation of the charge carries throughout the matrix due to strong nanomaterial-PANI interfacial adhesion [13]. However, in most of the preceding studies, graphite or graphene aggregates instead of stable graphene dispersions were attained, which reduces the large specific surface area of these nanomaterials and is detrimental for improving the composite electrical properties. In this context, it is crucial to improve the graphene dispersion and its interfacial interaction with the matrix. Consequently, significant effort has been devoted to surface-functionalize graphene and its derivatives as well as to optimize the composite preparation procedures. For example, Xu et al. [14] developed a simple method to prepare hierarchical nanocomposites with aligned PANI nanowires coated with GO nanosheets via dilute polymerization. Wu et al. [15] designed a facile one-step environmentally friendly polymerization method for the preparation of PANI/rGO nanocomposites using dialdehyde starch as reducing and capping agent. Besides, Kumar et al. [16] fabricated PANI-grafted-rGO nanocomposites with high electrical conductivity by functionalizing GO with 4-aminophenol via acyl chemistry. In particular, GO, a graphene derivative that contains epoxides, hydroxyls and carbonyls on the basal planes and carboxylic acids on the edges, has great potential as filler to develop PANI nanocomposites. It presents very large specific surface area, excellent chemical versatility and stability, aqueous processability, amphiphilicity, surface functionalization capability and biocompatibility [17]. More importantly, it can form stable aqueous colloids to enable the assembly of macroscopic structures, which is key for large-scale applications.

In a preceding work [18], we have developed a simple approach to functionalize GO via reaction with organic hexamethylene diisocyanate (HDI) using triethylamine (TEA) as catalyst. The synthesized (HDI)-modified GO nanomaterials, with different functionalization degrees, were found to be more hydrophobic in nature than pristine GO and were easily dispersed in polar aprotic solvents, hence are perfect candidates as fillers of conducting polymers. In the present study, we report a novel method to prepare PANI nanocomposites with homogenously dispersed GO nanosheets via in situ polymerization of aniline in the presence of HDI-GO using ammonium peroxydisulfate as an oxidizing agent in acid medium. HDI-GO can interact with PANI via electrostatic, hydrophobic, π-π stacking and hydrogen bonding interactions, which result in strong polymer-nanomaterial interfacial adhesion. Enhancing the dispersion of GO and its interaction with the conducting polymer matrix is decisive to attain improved electrical, thermal and mechanical properties. The resulting PANI/HDI-GO nanocomposites have been characterized by scanning electron microscopy (SEM), Fourier-transformed infrared spectroscopy (FT-IR), Raman spectroscopy, X-ray diffraction (XRD) analysis, thermogravimetric analysis (TGA), tensile tests and sheet resistance measurements to get information about how the weight fraction and functionalization degree of HDI-GO influence the nanocomposite morphology, structure and properties. This work signifies a great step forward to developing highly conducting PANI-based nanocomposites for application in a variety of organic devices.

## 2. Materials and Methods 

### 2.1. Reagents

Aniline monomer (C_6_H_5_NH_2_, >99%, *M*_w_ = 93.13 g/mol, d_25ºC_ = 1.02 g/cm^3^), ammonium persulfate ((NH_4_)_2_S_2_O_8_, 98%, *M*_w_ = 228.20 g/mol, d_25ºC_ = 1.98 g/cm^3^), triethylamine (TEA, >98%, *M*_w_ = 101.193 g/mol), H_2_SO_4_, KMnO_4_, P_2_O_5_, K_2_S_2_O_8_ and H_2_O_2_ (30 wt % in water) were obtained from Sigma-Aldrich (Madrid, Spain). Graphite powder was purchased from Bay Carbon, Inc. (Bay City, MI, USA). Hexamethylene diisocianate (HDI, >99%, *M*_w_ = 168.196 g/mol) was provided by Acros Organics (Madrid, Spain). The organic solvents were HPLC grade and were purchased from Scharlau S.L. (Barcelona, Spain). Deionized water was obtained from a Milli-Q-Water-Purification-System (Millipore, Milford, DE, USA). Aniline was stored in a refrigerator and was distilled under reduced pressure prior to use; the rest of the chemicals were used as received.

### 2.2. Synthesis of Hexamethylene Diisocyanate-modified Graphene Oxide (HDI-GO)

HDI-GO was prepared according to the procedure described in our preceding works [18,19]. In short, firstly GO was synthesized using a modified Hummers´ method from flake graphite using H_2_SO_4_, K_2_S_2_O_8_, P_2_O_5_ and KMnO_4_ as oxidants. Secondly, GO was functionalized by weighing the necessary amount of the synthesized GO powder and placing it into a round-bottom flask, followed by addition of dried toluene under inert atmosphere. In a first trial, GO dispersion was sonicated in an ultrasonic bath for 2 h, and subsequently TEA catalyst and HDI reagent were added dropwise (GO:HDI:TEA weight ratio of 1:1:1); the mixture was then heated to 60 °C and stirred for 12 h under inert atmosphere. The resulting product (HDI-GO 1) exhibited a functionalization degree (FD) of 12.3% as measured by elemental analysis [18]. In a second synthesis, the procedure was similar but the GO dispersion was first ultrasonicated with a tip (3 probe sonication cycles with 5 min of break between cycles at 40% amplitude) followed by the 2 h of bath sonication. The resulting product (HDI-GO 6) had a FD of 18.1 %. 

### 2.3. Synthesis of PANI/HDI-Modified GO Nanocomposites

**Scheme 2:**
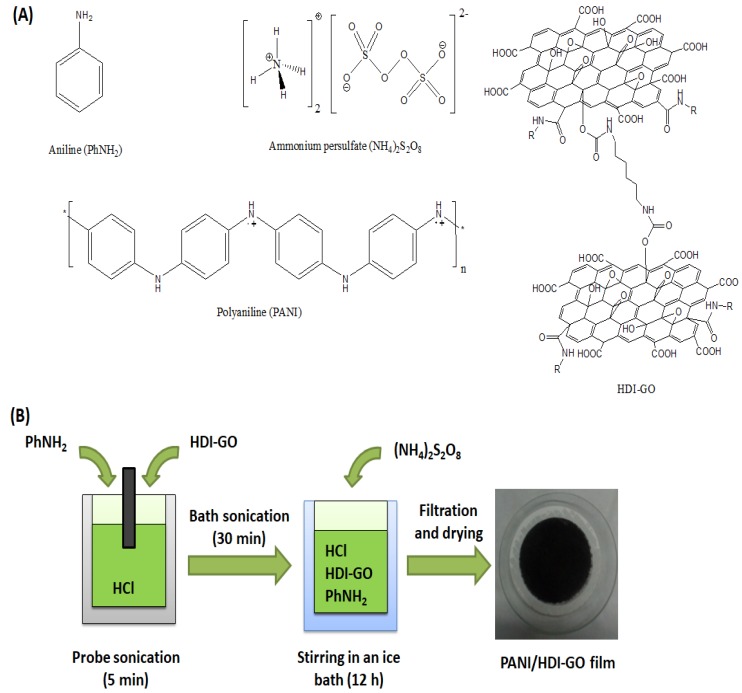
(**a**) Structure and nomenclature of the compounds used for the preparation of PANI/HDI-GO nanocomposites. (**b**) Diagram of the synthesis procedure of the nanocomposites.

The nanocomposites were prepared via *in situ* polymerization of aniline in acid medium, using (NH_4_)_2_S_2_O_8_ as oxidizing agent. The structure and nomenclature of the compounds used for the preparation of the PANI/HDI-GO nanocomposites is shown in Scheme 2a. 

Firstly, 0.1 mL of aniline monomer were placed in a beaker with 25 mL of 1 M HCl, and then the required amount of HDI-GO (0.25, 0.5, 1, 2.5 or 5 mg) was added. The mixture was then sonicated with an ultrasonic tip for 5 min and then in an ultrasonic bath for 30 min. Subsequently, 0.06 g of (NH_4_)_2_S_2_O_8_ were dissolved in 25 mL of 1 M HCl and then added dropwise to the aniline/HDI-GO mixture under vigorous stirring. Polymerization of aniline started after about 5 min, while the colour of the mixture changed into green. The mixture was then allowed to stir while cooled in an ice bath for 12 h.

The resulting solid precipitates, with HDI-GO weight ratios of 0.5, 1.0, 2.0, 5.0 and 10 wt %, were recovered from the reaction vessel by filtration and repeatedly washed with deionized water, ethanol, and hexane for the elimination of the low molecular weight polymer and oligomers until the filtrates were colourless, and finally dried at 80 °C in an oven for 48 h. A schematic representation of the synthesis procedure of the nanocomposites is shown in Scheme 2b. For comparative purposes, a reference sample containing 5 mg of pristine GO (10 wt % loading) and a reference PANI sample (0 wt % HDI-GO) were prepared in a similar way. Further, to investigate the influence of the level of functionalization of HDI-GO on the nanocomposite properties, two sets of composites were prepared, using HDI-GO 1 and HDI-GO 6, respectively. Photographs of the nanocomposites obtained are shown in Figure S1 in the supplementary material. Homogenous films were also prepared by dropping 5 mL of the liquid nanocomposite mixture onto a glass surface and dried in the oven at 80 °C.

### 2.4. Intrumentation 

A Selecta 3001208 ultrasonic bath (Madrid, Spain) and a 24 kHz Hielscher UP400S ultrasonic tip (Teltow, Germany) with a maximum power output of 400 W, equipped with a titanium sonotrode (Ø = 7 mm, l = 100 mm) were used to prepare the different dispersions.

The morphology of the nanocomposites was analyzed with a SU8000 Hitachi scanning electron microscope (SEM, Hitachi, Ltd., Tokyo, Japan), which operated with an emission current of 10 mA and a 15.0 kV accelerating voltage. Before the experiments, samples were coated with a 5 nm thick Au:Pd overlayer to avoid charge buildup throughout electron irradiation.

Raman spectra were acquired at room temperature with a laser output power of 1 mW using a Renishaw Raman microscope (Gloucestershire, UK) incorporating a He-Ne laser (632.8 nm). To minimize the signal-to-noise ratio, 3 scans were recorded for each composite. Data were then processed with the WiRE 3.3 Renishaw software, and the spectra were normalized to the G band for the sake of comparison. 

A TA Instruments Q50 thermobalance (Barcelona, Spain) was used to analyze the thermal stability of the samples via thermogravimetric analysis (TGA) experiments under an inert atmosphere (N_2_). Measurements were performed from 100 to 700 °C at a heating rate of 10 °C/min, with a gas flow rate of 60 mL/min. Prior to the tests, samples were dried for 72 h and then located inside aluminum pans.

The zeta potential of aqueous dispersions of the samples was measured at 25 °C on a Zetasizer Nano ZS (Malvern Instruments Ltd, Worcestershire, UK) using disposable plastic cuvettes.

X-ray diffraction (XRD) analysis was carried out with a Bruker D8 Advance diffractometer (Karlsruhe, Germany) using a Cu tube as X-ray source (λ CuKα = 1.54 Å), with a voltage of 40 kV and an intensity of 40 mA.

A standard test method for tensile properties of thin plastic sheeting, ASTM D882, was used for testing the mechanical properties of the composites. Tensile testing was carried out with an Instron 5565 Testing Machine (Norwood, MA, USA). A 1 kN load cell was used and the crosshead speed was 10 mm min^−1^. The results reported are the mean values for six replicates. 

A four-point probe resistivity measurement system (Multiheight Probe station, Leighton Buzzard, UK) was used to determine the sheet resistance (R_s_) of the samples. The voltage was measured with a KEITHLEY 2182A nanovoltmeter and the electrical current with a KEITHLEY 6221 current source. R_s_ was calculated as: R_s_ = 4,532 × (V/I), being V the test voltage and I the current. To ensure reproducibility, more than 5 measurements were carried out at different points of each sample film, and the results reported correspond to the average values.

## 3. Results

### 3.1. Morphology of PANI/HDI-GO Nanocomposites

**Figure 1:**
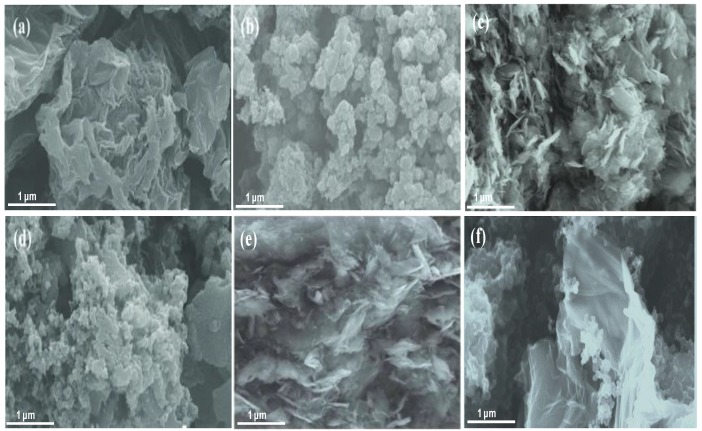
Representative SEM micrographs of raw GO (**a**), neat PANI (**b**), HDI-GO 6 (**c**), PANI/HDI-GO 6 (10 wt %) (**d**), HDI-GO 1 (**e**), and PANI/HDI-GO 1 (10 wt %) (**f**).

The morphologies and structures of PANI, GO, HDI-GO and the nanocomposites were studied by SEM, and some representative micrographs are shown in Figure 1. A granular or globular–like morphology is observed for pure PANI, comprising highly aggregated particles (Figure 1b). This is the most common structure found in PANI prepared by precipitation polymerization when using strong oxidants and acidic conditions [20]. Raw GO also appears rather compact and aggregated (Figure 1a), comprising wrinkly and flexible graphene sheets with thicknesses in the range of 10–50 nm. On the other hand, HDI-GO 6 is composed of stacked graphene flakes with a smoother surface topology (Figure 1c), ascribed to the covalent bonding of the HDI chains onto the GO surface. Further, many sheets appear stiffer, likely due to the wrapping of the methylene groups that cover the wrinkles, hence come out thicker, showing flake thicknesses ranging from 20 to 80 nm. Nonetheless, it should be noted that the sample appears quite heterogeneous, which is reasonable considering that it is a mixture of raw GO sheets and HDI modified sheets. Similar morphology was found for HDI-GO 1 (Figure 1e), albeit with thinner and less rigid sheets, suggesting that the flake stiffness and thickness rise with increasing FD. 

**Scheme 3:**
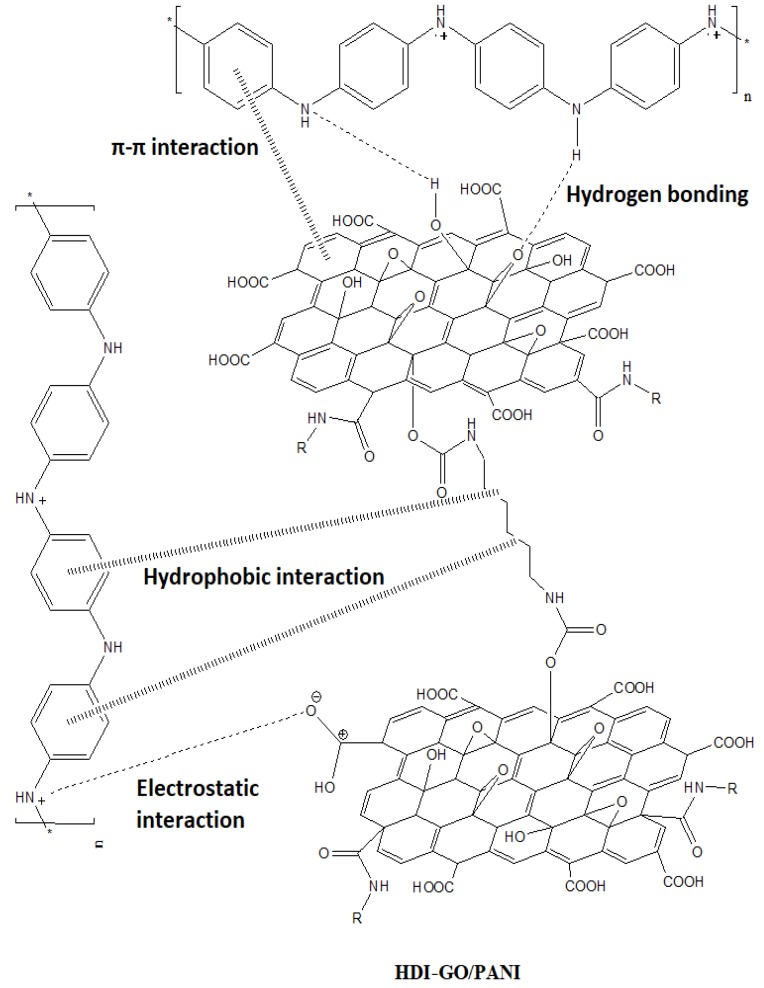
Representation of the different types of interactions between PANI and HDI-GO.

Focusing on the nanocomposite with 10 wt % HDI-GO 6 (Figure 1d), it can be observed that the flat and rigid graphene nanosheets are fully embedded within the PANI nanoparticles, forming a dense and interpenetrating network with the matrix particles partly attached onto the HDI-GO surface. It has been reported [21] that when HCl is used as a dopant, aniline monomer adsorbs onto the surface of graphene-based nanomaterials via electrostatic attraction, resulting in the formation of weak charge-transfer complexes between the polymer monomers and the graphitic structure of graphene. In our study PANI should adhere more strongly to the graphene flakes since some of the residual carboxylic groups of HDI-GO are negatively charged due to resonance effects, hence are prone to strongly interact with the positively charged emeraldine salt form of PANI via electrostatic interactions; further, the aromatic moieties of the polymer can interact with the benzene rings of HDI-GO via π-π stacking as well as with the methylene chains of the HDI through hydrophobic interactions. Besides, hydrogen bonding can occur between the amine groups of PANI and the oxygenated functional moieties of HDI-GO (see Scheme 3). Thus, the combination of all these interactions results in strong PANI-HDI-GO interfacial adhesion.

As a result of the absorption process, the HDI-GO nanosheets were coated by the matrix particles during the in situ polymerization reaction in acid medium. This allowed to prepare homogeneous composites in which PANI and the modified GO are likely intercalated with each other instead of individually being in an agglomerated state and phase separated as typically observed previously [22,23]. The lessening in the strength of the hydrogen bonding interactions among the GO nanosheets upon functionalization with HDI makes the HDI-GO surface more hydrophobic than that of raw GO [18], henceforth it was well dispersed in HCl by the combination of probe and bath sonication used herein. Thus, PANI chains were able to diffuse within the stacked structure of HDI-GO and formed a thin coating onto the graphene nanosheets. This morphology was retained for all the composites reinforced with HDI-GO 6, which showed a relatively smooth and plain surface, and the degree of interpenetration increased with increasing the nanomaterial loading. Another plausible explanation for the observed morphology could be the formation of intimate charge-transfer complexes in the solid state due to very strong donor-acceptor interactions between the polymer and the nanofiller, as reported previously for PANI/rGO nanocomposites [24]. Thus, HDI-GO could act as a stable counterion of the doped state of PANI emeraldine salt, stabilizing PANI in an intermediate oxidation state between the leucoemeraldine and the emeraldine forms. In addition, this charge-transfer in PANI/HDI-GO nanocomposites together with the numerous graphene bundles randomly distributed throughout the matrix would be beneficial for the formation of homogenous conductive paths, and would account for the improved nanocomposite properties including improved electrical conductivity and superior thermal stability, as will be discussed in the following sections. 

Conversely, in the nanocomposite with 10 wt % HDI-GO 1 (Figure 1f) there is lack of such interpenetrating polymer-nanofiller network, and both composite components appear much more separated. During the in situ polymerization process, the PANI nanoparticles located within the GO layers and acted as spacers to form gaps between neighboring GO sheets, thus resulting in flakes with a large surface area and high level of exfoliation that retain their bendability and flexibility. Nonetheless, some flakes also display a random decoration of PANI nanoparticles over the wrinkled surface. Similar morphology was found for the rest of nanocomposites incorporating HDI-GO 1, with increasing number of decorated nanosheets with increasing nanofiller loading. 

### 3.2. XRD Analysis of the Nanocomposites

**Figure 2:**
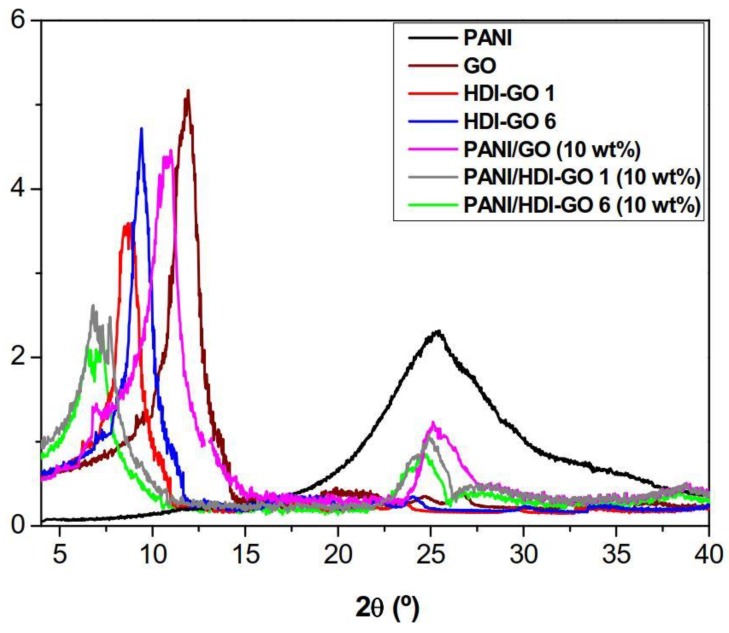
XRD patterns of neat PANI, raw graphene oxide (GO), hexamethylene diisocyanate-modified GO (HDI-GO) 1, HDI-GO 6, and the corresponding nanocomposites with 10 wt % nanofiller content.

The developed samples were analyzed by XRD measurements, and typical patterns of PANI, GO, HDI-GO 6, HDI-GO 1 and the corresponding nanocomposites with 10 wt % loading are compared in Figure 2. Analogous diffractograms were found for the rest of PANI/HDI-GO nanocomposites, and the data obtained from the diffraction patters are collected in Table S1 (supplementary material). Neat PANI shows a broad peak centered at 2θ = 25.3º, indicative of its amorphous character and the absence of order arrangement in the polymer chains. This wide peak can also be observed in the diffractograms of the nanocomposites, although appears at lower 2θ values; further, its intensity decreases with increasing nanofiller loading, in agreement with the results reported previously for PANI/GO hidrogels [25]. The presence of GO reduces the percentage of PANI in the nanocomposites and hence weakens the diffraction peaks.

The downshift in 2θ is more pronounced for nanocomposites reinforced with HDI-GO compared to that with GO (see Table S1), in particular for those with HDI-GO 6, the utmost decrease being 0.79° for PANI/HDI-GO 6 with 10 wt % loading. This shift in 2θ towards lower values has been previously reported for PANI/rGO nanocomposites [26], indicative of an expansion of the interlayer distance, and can be explained by the adsorption and intercalation of PANI on the surface and between the HDI-GO sheets. Thus, as the functionalization degree of HDI-GO increases, the stronger the interactions with the matrix chains, the stronger the adsorption of PANI, the more pronounced the shift in the peak maximum, in agreement with the observations from SEM study (Figure 1). Therefore, the absorption and intercalation of PANI on the GO nanosheets seems to be responsible for the nanocomposites structure and morphology. 

Regarding pristine GO, a characteristic peak can be observed at 2θ = 11.8° related to the (002) diffraction of the GO sheet [27], from which the interlayer *d* spacing value was calculated to be 0.748 nm according to the Bragg’s equation [28]. In the case of HDI-GO 1 and HDI-GO 6, the peak maximum appears at lower 2θ (9.2 and 8.8°, corresponding to interlayer distances of 0.961 and 1.003 nm, respectively) and shows reduced intensity. This increase in the *d* spacing has been previously observed for other nanocomposites comprising polymer chains between GO nanosheets [29,30], and further corroborates the presence of the HDI segments intercalated between the nanofiller layers. 

Regarding the nanocomposites, further downshift of this (002) peak is observed, suggesting an additional rise in the interlayer spacing of the carbon nanomaterial compared to either GO or HDI-GO. In the case of PANI/GO (10 wt %), the downshift is relatively small, about 1° compared to raw GO, resulting in a *d* spacing of ~0.8 nm. In contrast, nanocomposites reinforced with 10 wt % HDI-GO 1 and HDI-GO 6 show stronger shifts, and exhibit *d* values of 1.18 and 1.33 nm, respectively (Table S1). These clear increases in the interlayer spacing are indicative of the strong interaction between the PANI segments and the GO nanosheets, and the intercalation of the polymeric chains in between the nanomaterial layers. Once more, the higher the FD of HDI-GO, the larger the interlayer distance in the nanocomposites. The broadening of the (002) peak is also evident for all the PANI/HDI-GO compositions compared to either HDI-GO 1 or HDI-GO 6. Thus, the width at half maximum increased from about 1.4° for HDI-GO 6 to 2.9° for the nanocomposite with 10 wt % loading. This widening is yet another indication of the strong PANI-HDI-GO 6 interactions. The combination of the substantial shift of the (002) peak during the formation of PANI/HDI-GO and the reduction in the GO peak height intensity further corroborate the formation of hybrid intercalated nanocomposites, as observed previously [31]. On the whole, the XRD patterns confirm the efficiency of the in situ polymerization process used in this study to facilitate the diffusion of the PANI chains within the interlayer spacing of the HDI-GO nanosheets. 

### 3.3. Raman Spectra 

**Figure 3:**
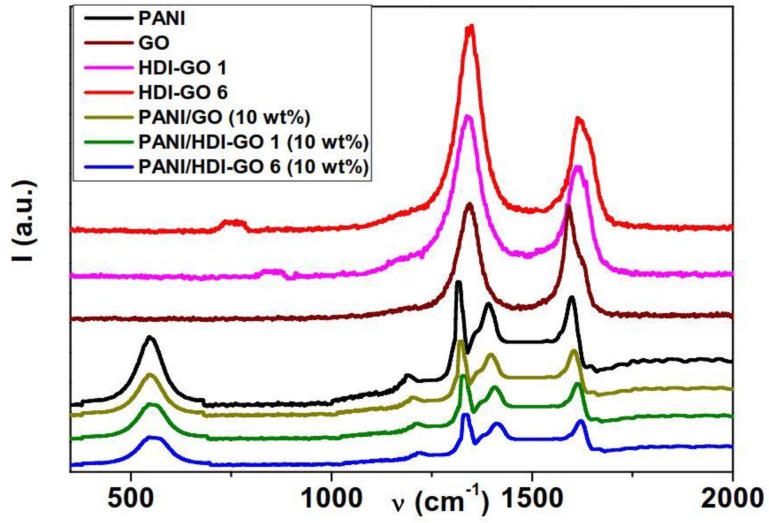
Raman spectra of neat PANI, raw GO, HDI-GO 1, HDI-GO 6, and the corresponding nanocomposites with 10 wt % nanofiller content.

Raman spectroscopy was used to further characterize PANI and the synthesized nanocomposites, and the spectra obtained for neat PANI, GO, HDI-GO, and the nanocomposites with 10 wt % HDI-GO or GO are shown in Figure 3. Data derived from the spectra of all the nanocomposites are collected in Table S1 (supplementary material).

Characteristic Raman peaks of PANI can be observed within the wavelength range 400–1800 cm^-1^. The C=N stretching vibration in the quinonoid units of PANI is observed at 1396 cm^−1^ [32]. The band at 1191 cm^−1^ corresponds to the C–N stretching vibrations of benzenoid, quinonoid, and polaronic forms of PANI in the emeraldine state, and the presence of C–N^+^ vibrations are evident at 1390 cm^−1^ [32,33]. The band at 1597 cm^−1^ corresponds to the C=C stretching of the benzenoid ring vibrations, and that at 555 cm^-1^ is attributed to C–H in plane bending vibrations [33]. Regarding GO, the most prominent features in the spectrum are the disorder induced D band at 1345 cm^−1^ that indicates the level of structural disorder, and the tangencial G band at ~1595 cm^−1^ related to in-plane displacements of the graphene nanosheets [34]. Analogous spectrum is observed for the HDI-GO samples, although they show an enlargement and upshift of the G band, attributed to a change in the electronic structure of GO in the presence of electron-acceptor groups [18]. This upshift can also be associated to a raise in defect density, given that the position of the G band depends on the concentration of defects in the graphene layers [35]. Further, the D to G band intensity ratio (*I*_D_/*I*_G_) offers quantitative information about the amount of defects in graphene sheets: The higher the ratio, the larger the disorder [35]. This ratio is 1 for neat GO, and increases up to 1.55 and 1.73 for HDI-GO 1 and HDI-GO 6, respectively, which corroborates the drop in structural order upon anchoring the HDI chains onto the GO surface. 

Focusing on the nanocomposites, the spectra are analogous to that of neat PANI, albeit they exhibit an upshift in the position of the peaks as well as a drop in the intensity of the bands. In the case of PANI/GO (10 wt %), the shift in the band positions is relatively small, indicative of weak interactions between the PANI chains and GO (Table S1). However, PANI/HDI-GO 1 (10 wt %) and PANI/HDI-GO 6 (10 wt %) show strong shifts in the position of the bands, by up to 15 and 26 cm^−1^ in the C=C stretching of the benzenoid ring. This behavior is yet another confirmation of the strong adsorption of the PANI chains onto the GO or HDI-GO surface via π-π stacking, H-bonding, hydrophobic and electrostatic interactions, leading to the formation of a tightly coated PANI layer on the carbon nanomaterial surface, as discussed previously. Thus, the combination of all these interactions results in strong PANI-HDI-GO interfacial adhesion. Moreover, the higher the FD of HDI-GO, the stronger the interactions with the matrix chains, hence the larger the change in the position of the peaks. Thus, the upshifts in the position of the peaks are systematically larger for composites with HDI-GO 6 compared to those with the same loading of HDI-GO 1. An upshift in the position of PANI peaks corresponding to C=N and C-C stretching modes has also been reported for PANI nanofibers coated with graphene nanosheets, indicating the very intense interaction between the nanofibers and the carbon nanomaterial in the composite [13].

### 3.4. Thermal Stability of the Nanocomposites

**Figure 4:**
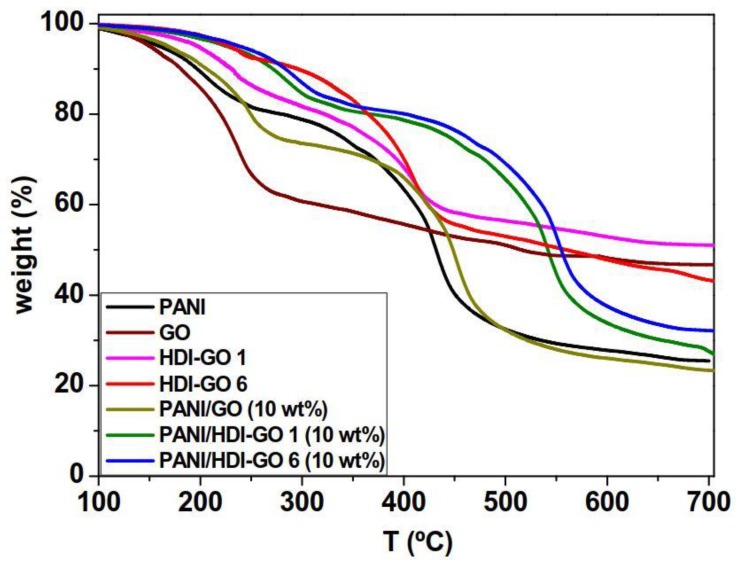
Thermogravimetric analysis (TGA) curves under inert atmosphere of neat PANI, GO, HDI-GO 1, HDI-GO 6, and the nanocomposites with 10 wt % nanofiller loading.

A high thermal stability of nanocomposite films is a desirable factor for certain applications like electromagnetic shielding (EMI) at high temperatures. TGA analysis upon heating under a nitrogen atmosphere was carried to examine the thermal stability of PANI and the PANI/GO nanocomposites (Figure 4). The results derived from all the nanocomposites are collected in Table S2 (supplementary material). Regarding pure PANI, the weight loss below 150 °C can be assigned to the loss of water molecules and other volatile impurities. The first main decomposition stage between 150 and 250 °C is attributed to the deprotonation of PANI through removal of dopant HCl molecules [33], while the second from 340 to 480 °C can be assigned to the exothermic thermal decomposition of PANI backbone. In the case of pristine GO, a one-step degradation process is found, with a main weight loss below 250 °C attributed to the decomposition of surface epoxide, hydroxyl, and carboxylic acid functional groups [18]. In addition, a small weight loss is observed above 250 °C due to the elimination of further functional groups. On the other hand, the HDI-GO samples display two decomposition steps, the first due to the removal of remaining oxygenated surface groups, and the second to the degradation of the HDI chains linked to the GO surface. With increasing FD, the degree of crosslinking between the GO layers increases, hence the thermal stability of HDI-GO improves [18].

The nanocomposites also exhibit a two-step degradation process, similar to that of neat PANI, albeit shifted to higher temperatures, indicating a thermal stabilization effect induced by the presence of the nanofillers. Nonetheless, the shift depends on the type of nanofiller and its concentration. Thus, upon addition of 10 wt % GO, the initial degradation temperature (T_i_) at 2% weight loss increased only slightly (about 12 °C), whereas by addition of the same amount of HDI-GO 1 and HDI-GO 6, it increased significantly, by 53 and 64 °C , respectively. Similar trend is found for the temperature of 10% weight loss (T_10_) and the temperatures of maximum rate of weight loss of both stages (T_maxI,II_), which show maximum augments of 67 and 90 °C for the nanocomposite with 10 wt % HDI-GO 1 and increments of up to 81 and 109 °C for that with 10 wt % HDI-GO 6, respectively (Table S2). In addition, the weight residue of PANI/GO (10 wt %) is slightly lower than that of neat PANI, whilst that of nanocomposites with 10 wt % HDI-GO 1 and HDI-GO 6 are about 10 and 27% higher, respectively. The extraordinary thermal stability improvements found in the nanocomposites reinforced with HDI-GO can be explained considering the homogenous dispersion of the nanosheets within the PANI chains, as revealed by SEM images (Figure 1d,f) combined with the strong PANI-HDI-GO interfacial adhesion via π-π stacking, H-bonding, hydrophobic and electrostatic donor-acceptor interactions that lead to the formation of intimate charge-transfer complexes in the solid state [24], as discussed earlier. The crosslinked HDI-GO layers, which are randomly dispersed within the polymer matrix, can behave as a barrier and a thermal protecting material that shield the PANI chains from the heat and delay the diffusion of the degradation products from the interior of the nanocomposite to the gas phase through the formation of a tortuous path. 

Similar behavior of thermal stability improvement has been reported for PANI nanocomposites reinforced with graphene nanoplatelets via in situ polymerization, attributed to the formation of 3D conducting interpenetrating networks between the polymer and the nanofillers [36]. Thus, in the nanocomposites comprising HDI-GO 6, the stiff graphene nanosheets are fully embedded within the PANI nanoparticles, forming a dense and interpenetrating network with the matrix particles partly attached onto the HDI-GO surface, which can account for the superior thermal stability enhancement observed. Further, the strong PANI:HDI-GO interactions likely confine the rotational movement of the polymeric chains, and the motion restriction of the polymeric chains at the PANI: HDI interface would also be reflected in better thermal stability, as reported for PANI nanofiber-coated polystyrene/GO nanocomposites [37]. The comparison of the degradation temperatures of nanocomposites reinforced increasing amounts of HDI-GO 1 or HDI-GO 6 (Table S2) reveals that all the degradation temperatures systematically increase with increasing HDI-GO loading, likely due to the stronger barrier effect imposed by the nanofillers (i.e., an average increase of 40 °C when the nanofiller loading is increased from 0.5 to 10 wt %). TGA data reveal that the addition of HDI-GO with a high FD strongly improves the thermal stability of PANI, which is an important result from a practical viewpoint including aerospace, electronic and photovoltaic applications.

### 3.5. Sheet Resistance of the Nanocomposites

**Figure 5:**
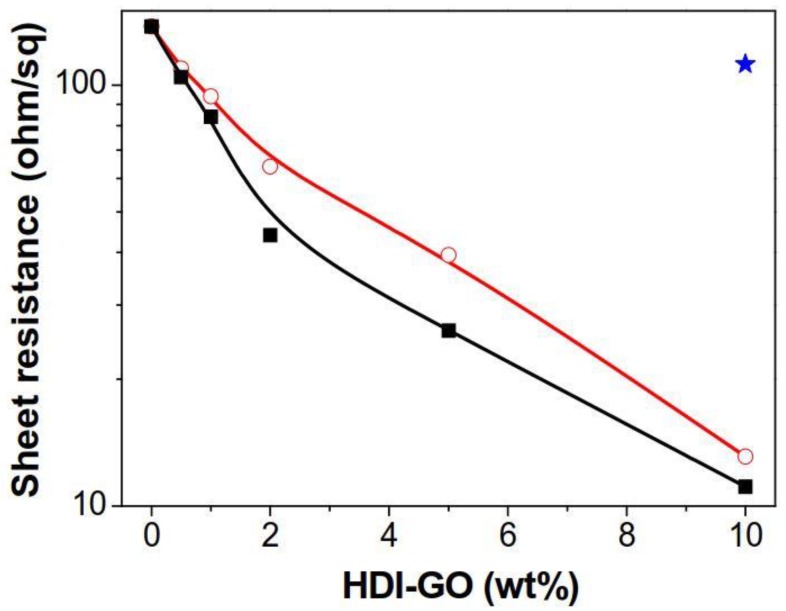
Sheet resistance of PANI nanocomposites reinforced with HDI-GO 6 (solid squares) and HDI-GO 1 (open circles) as a function of the HDI-GO content. Solid lines are guides for the eyes. The blue star corresponds to the PANI/GO (10 wt %) reference composite.

Figure 5 shows the sheet resistance (*R*_s_) data of neat PANI and the nanocomposites reinforced with different amounts of HDI-GO 1 and HDI-GO 6. The neat PANI film shows an *R*_s_ value close to 135 Ω/sq., which drops steadily with increasing HDI-GO loading, leading to minimum values of 11 and 13 Ω/sq for the nanocomposites reinforced with 10 wt % HDI-GO 6 and HDI-GO 1, respectively. However, the reference PANI/GO sample (blue star in the plot) showed similar sheet resistance to that of PANI. Taking into account that the HDI-GO samples display higher R_s_ than neat PANI [19], the reduction in sheet resistance found in PANI/HDI-GO nanocomposites is a surprising behavior that could be explained considering the different factors that influence the charge hopping conduction mechanism in this type of conductive polymer, including grain size, level of crystallinity, doping and screening effects as well as conformational changes of the polymeric chains [38]. 

The conduction mechanism is believed to involve polaronic carriers. When the emeraldine base is doped in an acid environment, a polaron or bipolaron can be developed via consecutive formation of positive species. Bipolaron structures are thermodynamically more stable and conductive, and are responsible for the electrical conduction via a jump mechanism [2]. The adjacent nitrogen electron (neutral) moves to a vacant spot and neutralizes it. Consequently, this spot moves, thereby creating new spaces in the nitrogen structure and in the polarons structures, resulting in electron transportation and, thus, electrical conductivity along the chain [38]. 

The structure, morphology and synthesis method of the nanocomposites has also a profound effect on the electrical conductivity of the materials [39]. Thus, the presence of GO poorly dispersed within PANI has been reported to decrease the conductivity of the polymer [40], while that of composites prepared via in situ emulsion polymerization was significantly improved [41], ascribed to the very homogeneous nanofiller dispersion inside the polymer matrix that made both composite components come close to each other to form conductive paths. Further, a noticeable electrical conductivity improvement was previously reported for PANI/graphene nanoplatelets developed via in situ polymerization, attributed to the formation of 3D conducting interpenetrating networks [36].

Further, our Raman spectra suggest the presence of strong π-π stacking, H-bonding, hydrophobic and electrostatic interactions, in particular for the composites with HDI-GO 6, leading to the formation of a tightly coated PANI layer on the carbon nanomaterial surface. Besides, as mentioned earlier, the HDI-GO could behave as a counterion of the emeraldine salt form of PANI, stabilizing it in an intermediate oxidation state between the leucoemeraldine and the emeraldine structures. This would result in the formation of tight PANI-HDI-GO charge-transfer complexes via strong donor-acceptor interactions, hence better charge carrier transport, and consequently reduced sheet resistance for the nanocomposites. To further corroborate the HDI-GO coverage by the PANI chains, the zeta potential (**ζ)** of aqueous dispersions of GO, PANI, HDI-GO 1, HDI-GO 6 and their composites with 10 wt % was measured, and the results are included in Table S2 (Supplementary Information). The emeraldine form of PANI carries positive charges, which make it dispersible in aqueous solution, leading to a positive zeta potential of 40 mV, consistent with the results reported previously [42]. **ζ** of GO is highly negative, due to the large number of surface oxygenated functional groups on the graphene sheets. However, **ζ** of HDI-GO is significantly smaller (in absolute value), albeit it is still negative, indicative of the presence of remaining oxygen containing groups on the graphene sheets, in agreement with the results from elemental analysis [18]. As expected, with increasing FD, **ζ** value decreases. Moreover, as compared to the negatively charged GO, all the PANI/GO nanocomposites are positively charged, which indicates coverage of PANI on the graphene surface. The electrostatic interaction between the positively charged PANI and the negatively charged nanofillers is stronger in composites with HDI-GO (particularly those with HDI-GO 6) compared to the reference with GO, hence leading to a lower **ζ**. Further, with increasing HDI-GO content, the interaction becomes more pronounced, thus **ζ** decreases. 

It is noteworthy that the PANI/HDI-GO samples prepared in this work display higher conductivity than that previously reported for other PANI-GO nanocomposites [40,43,44], which demonstrates the effectiveness of the strategy developed in this study to improve the thermoelectrical properties of conductive polymer/graphene nanocomposites. Further, the values obtained are also higher than those found for PANI/multiwalled carbon nanotube (MWCNT) nanocomposites, likely due to the larger surface area of HDI-GO nanosheets compared to the nanotubes [45]. For the same nanofiller content, R_s_ is systematically lower for nanocomposites filled with HDI-GO 6 compared to those with HDI-GO 1. This can be rationalized considering that HDI-GO 6, with a higher FD, is more homogeneously dispersed within the matrix, and interacts more strongly with the PANI chains, thus favoring the formation of a more interpenetrated network, and consequently conductive paths, hence lower sheet resistance. It is interesting to note that the nanocomposites with high HDI-GO content exhibit R_s_ values close to those reported for ITO films coated onto plastic substrates, such as polyethylene terephthalate (PET) [46], hence, they are good candidates to be used as transparent conductive electrodes in conventional panel displays, solar cells, touch panels, and so forth. 

### 3.6. Mechanical Properties

**Figure 6:**
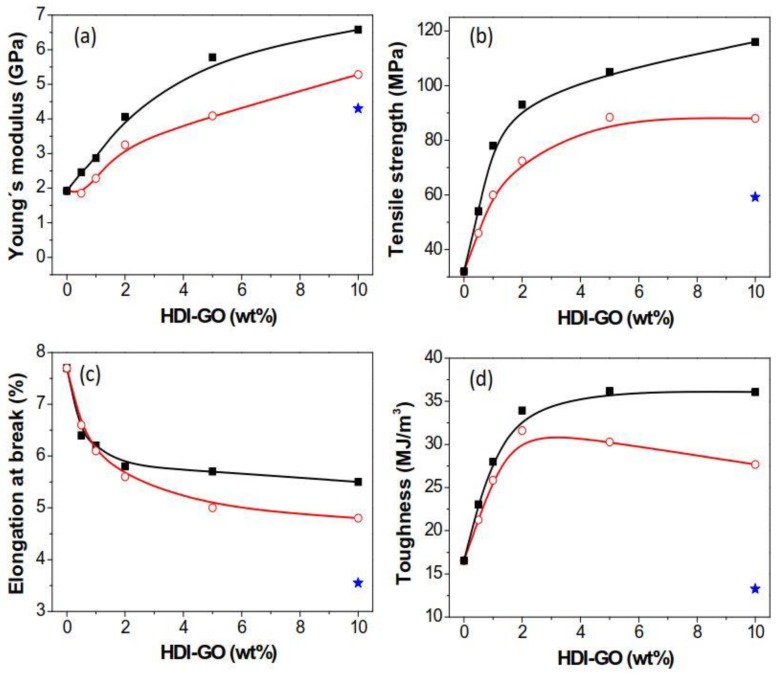
Results derived from tensile tests of PANI nanocomposites reinforced with HDI-GO 6 (solid squares) and HDI-GO 1 (open circles) as a function of the HDI-GO content. (**a**) Young’s modulus; (**b**) tensile strength; (**c**) elongation at break; (**d**) toughness. Solid lines are guides for the eyes. The blue star corresponds to the PANI/GO (10 wt %) reference composite.

The mechanical properties of PANI/HDI-GO nanocomposites were investigated by tensile tests, and the stress-strain curves of PANI and some PANI-based nanocomposites are shown in Figure S2 in the supplementary material; the results derived from the tensile curves of all the samples tested are depicted in Figure 6. Neat PANI exhibits an elastic modulus close to 2 GPa and a tensile strength value of about 32 MPa, in very good agreement with the data reported earlier [47]. The addition of HDI-GO 6 causes significant enhancements in both parameters (Figure 6a,b), by up to 240 and 258%, respectively, at the highest loading tested. A similar tendency, although with smaller increases, can be observed for the nanocomposites comprising HDI-GO 1. For both types of nanocomposites, the rise is more pronounced up to 2 wt % loading, whilst it is less marked at higher concentrations, and appears to level off at HDI-GO contents > 10 wt %. At low nanofiller weight fractions, there would be larger number of individual HDI-GO nanosheets, hence the reinforcing capability would be stronger than that found at higher loadings, since the HDI-GO nanosheets are more stacked. The modulus and strength improvements attained herein are higher than those reported previously for PANI/graphene composites, corroborating the high reinforcing efficiency of HDI-GO, in particular that with the highest FD, probably due to the combination of a random and very homogenous nanomaterial dispersion within the matrix and a very strong PANI-HDI-GO interfacial adhesion attained via hydrogen bonding, electrostatic, hydrophobic, and π-π stacking interactions, as mentioned above (Scheme 3), together with the high modulus of GO (~207 GPa [48]). The enhancements are also greater than those reported for PANI/MWCNT composites [49], despite the higher modulus of MWCNTs compared to GO, suggesting improved nanofiller-matrix stress transfer in the nanocomposites with HDI-GO nanosheets. The improvements in modulus and strength for the reference PANI/GO (10 wt %) nanocomposite were smaller, around 115 and 85%, respectively, likely due to the presence of aggregates (Figure 1a) that reduce the GO-PANI interfacial area and restrict the load transfer efficiency, combined with the weaker GO-PANI interactions. Good nanofiller dispersion and strong interfacial adhesion to the PANI matrix is the key point to improve the mechanical properties. 

Regarding the elongation at the break (Figure 6c), PANI presents a value close to 7.8%, which decreases with increasing HDI-GO content, the drop being more pronounced at lower loadings. The maximum drop is found to be 36% for the composite with 10 wt % HDI-GO 1. This is the typical trend found in nanofiller-reinforced polymer nanocomposites, since the fillers restrict the ductile flow of the polymer segments. Further, the strong PANI-HDI-GO interfacial adhesion attained by hydrogen bonding, electrostatic, hydrophobic and π-π interactions contributes to the reduced plasticity. The drop in ductility is in general more pronounced for the nanocomposites with HDI-GO 1 compared to those filled with HDI-GO 6, likely due to the presence of some nanofiller aggregates that restrict more strongly the mobility of the polymeric chains. On the other hand, the reference PANI/GO (10 wt %) nanocomposite shows the most drastic reduction in the elongation at break, about 55% compared to neat PANI, attributed to the larger number of aggregates. Nonetheless, the ductility of the developed nanocomposites is still reasonably good, and the combination of high strength and flexibility is interesting for applications in flexible electronics.

Regarding the toughness of the nanocomposites (the total energy required to break the composite estimated by the area under the stress-strain curve, Figure 6d), the trend observed is quite similar to that of the strength. The nanocomposite with 5 wt % HDI-GO 6 content shows the highest toughness, about 112% higher than that of neat PANI (16.9 MJ/m^3^). Beyond this concentration, the toughness remained roughly constant for nanocomposites comprising HDI-GO 6 while dropped slightly for those filled with HDI-GO 1. This trend differs from that found in the reference PANI/GO (10 wt %) nanocomposite that shows a toughness fall of about 28% compared to neat PANI, in agreement with the behavior typically observed in polymer/GO nanocomposites [4], in which the toughness is significantly reduced at high GO contents. The enhanced behavior found herein could be ascribed to the more homogeneous HDI-GO dispersion that avoids the formation of stress concentration points or crack initiators under applied loads, together with the stronger PANI-HDI-GO interfacial adhesion, that would result in a more effective barrier for the propagation of cracks. Further, according to SEM images (Figure 1), PANI and HDI-GO are intercalated with each other instead of individually being in an agglomerated state and phase separated as typically observed for other PANI/GO nanocomposites [22,23], which leads to a decrement in toughness. The simultaneous improvement in ductility and toughness is interesting from an application viewpoint, in particular for the development of flexible electronic devices. 

## 4. Conclusions

Multifunctional high-performance PANI-HDI-GO nanocomposites have been prepared via in situ polymerization of aniline in acid medium in the presence of HDI-GO as nanofillers and (NH_4_)_2_S_2_O_8_ as oxidizing agent. The morphology, structure, thermal stability, sheet resistance, zeta potential and mechanical properties of the nanocomposites have been characterized to elucidate the influence of the HDI-GO content and level of modification on the final properties. According to SEM observations, the flat and rigid HDI-GO nanosheets are fully embedded within the PANI nanoparticles, forming a dense and interpenetrating network with the matrix particles partly attached onto the nanomaterial surface. PANI can strongly interact with HDI-GO by means of hydrogen bonding, hydrophobic, electrostatic, and π-π stacking interactions, as confirmed by Raman spectroscopy and XRD analysis. TGA and four-point probe measurements reveal that the thermal stability and electrical conductivity of PANI, respectively, gradually rise with increasing HDI-GO loading, the improvement being more significant with increasing HDI-GO functionalization degree. The nanocomposites comprising 5 wt % HDI-GO with the highest modification level display an optimal balance of rigidity, strength, ductility and toughness. These results open novel opportunities for the development of multifunctional nanocomposites based on graphene derivatives and intrinsically conducting polymers to be used in a wide range of applications such as supercapacitors, sensing platforms, solar cells, fuel cells, electrochromic devices, lithium ion batteries, flexible plastics, wearable electronics and so forth.

## Supplementary Materials

The following are available online at https://www.mdpi.com/2073-4360/11/6/1032/s1, Figure S1: Photographs of PANI-based nanocomposites incorporating different GO or HDI-GO 6 contents. Figure S2: Stress-strain curves of PANI and some PANI-based nanocomposites. Table S1: Data obtained from the Raman spectra and XRD patterns of PANI/HDI-GO nanocomposites. Table S2: TGA and zeta potential data of PANI/HDI-GO nanocomposites.
