# Supplementary material for: The Effect of Hexamethylene Diisocyanate-Modified Graphene Oxide as a Nanofiller Material on the Properties of Conductive Polyaniline
Source: Polymers (Basel). 2019 Jun 11;11(6):1032. doi: 10.3390/polym11061032 (PMC6630901; doi:10.3390/polym11061032)
Supplement: Supplementary file 1 [file polymers-11-01032-s001.pdf]

Supplementary Materials

The Effect of Hexamethylene Diisocyanate-Modified Graphene Oxide as a Nanofiller Material on the Properties of Conductive Polyaniline

José Antonio Luceño Sánchez[1], Ana Maria Díez-Pascual[1]*, Rafael Peña Capilla[2] and Pilar García Díaz[2]

[1] Department of Analytical Chemistry, Physical Chemistry and Chemical Engineering, Faculty of Sciences, Alcalá University, 28805 Madrid, Spain; jose.luceno@uah.es

[2] Department of Signal Theory and Communication. Polytechnic High School, Alcalá University, 28805 Madrid, Spain; rafa.pena@uah.es, pilar.garcia@uah.es

*Correspondence: am.diez@uah.es; Tel.: +34-918-856-430

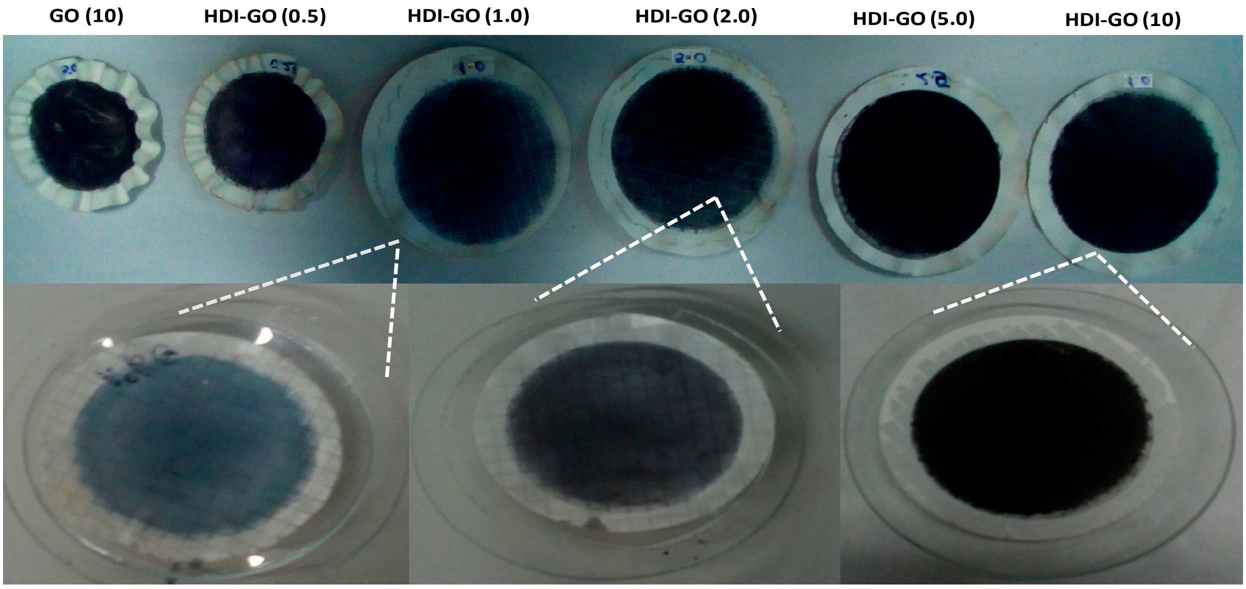

Figure S1. Photographs of PANI-based nanocomposites incorporating different GO or HDI-GO 6 contents.

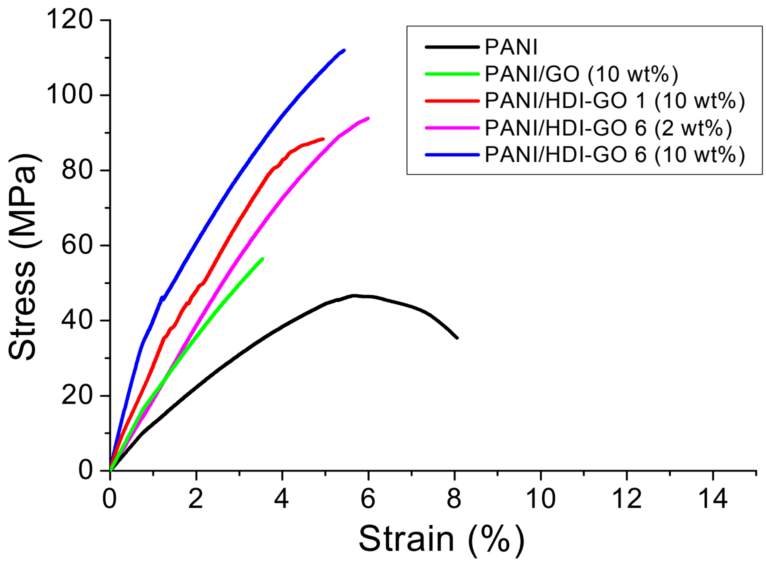

Figure S2. Stress-strain curves of PANI and some PANI-based nanocomposites.

Table S1. Data obtained from the Raman spectra and XRD patterns of PANI/HDI-GO nanocomposites.

Sample	ν_{C-N} (cm^{-1})	$\nu_{C=N}$ (cm^{-1})	$\nu_{C=C}$ (cm^{-1})	peak (2θ)[a]	I_{PANI}/I_{COM}[b]	d_{002}[c] (nm)
PANI	1191	1396	1597	25.31	–	–
PANI/GO (10 wt%)	1201	1399	1605	25.13	1.792	0.799
PANI/HDI-GO 1 (0.5 wt%)	1195	1398	1600	25.22	1.533	0.943
PANI/HDI-GO 1 (1.0 wt%)	1197	1400	1603	25.16	1.732	1.029
PANI/HDI-GO 1 (2.0 wt%)	1203	1403	1605	25.08	1.958	1.065
PANI/HDI-GO 1 (5.0 wt%)	1207	1405	1609	25.02	2.183	1.094
PANI/HDI-GO 1 (10 wt%)	1214	1407	1612	24.95	2.325	1.183
PANI/HDI-GO 6 (0.5 wt%)	1198	1401	1602	25.06	2.109	1.041
PANI/HDI-GO 6 (1.0 wt%)	1205	1404	1607	24.97	2.343	1.097
PANI/HDI-GO 6 (2.0 wt%)	1210	1408	1616	24.88	2.446	1.186
PANI/HDI-GO 6 (5.0 wt%)	1215	1411	1620	24.64	2.796	1.240
PANI/HDI-GO 6 (10 wt%)	1218	1412	1623	24.52	2.911	1.335

ν_{C-N}: C–N stretching vibrations of benzenoid, quinonoid, and polaronic forms of PANI; $\nu_{C=N}$: C=N stretching vibrations in the quinonoid units; $\nu_{C=C}$: C=C stretching of the benzenoid ring. [a]position of the peak maximum of PANI; [b]ratio of the intensity between the peak maximum of PANI and that of the composite; [c]interlayer *d* spacing values of (002) peak of GO.

Table S2. TGA and zeta potential data of PANI/HDI-GO nanocomposites.

Sample	T_i (ºC)	T_{10} (ºC)	$T_{max(I,II)}$ (ºC)	R (wt%)	ζ (mV)
PANI	123	193	199, 422	25.2	+40.1
GO	117	173	227	46.9	-38.6
HDI-GO 1	149	230	224,355	51.0	-23.7
HDI-GO 6	188	286	253,376	43.4	-18.5
PANI/GO (10 wt%)	135	204	229, 432	23.4	+34.6
PANI/HDI-GO 1 (0.5 wt%)	133	203	206, 430	25.0	+33.7
PANI/HDI-GO 1 (1.0 wt%)	146	217	217, 451	26.1	+28.9
PANI/HDI-GO 1 (2.0 wt%)	158	235	238, 574	26.7	+21.5
PANI/HDI-GO 1 (5.0 wt%)	166	254	244, 589	27.0	+17.6
PANI/HDI-GO 1 (10 wt%)	176	270	266, 512	25.5	+12.4
PANI/HDI-GO 6 (0.5 wt%)	135	207	211, 434	26.3	+28.3
PANI/HDI-GO 6 (1.0 wt%)	148	226	233, 457	27.8	+23.9
PANI/HDI-GO 6 (2.0 wt%)	162	245	257, 482	29.4	+19.3
PANI/HDI-GO 6 (5.0 wt%)	179	263	365, 512	30.9	+15.2
PANI/HDI-GO 6 (10 wt%)	187	283	280, 531	32.2	+9.7

T_i: initial degradation temperature at 2% weight loss; T_{10}: temperature of 10% of weight loss. T_{max}: temperature of maximum rate of weight loss. The subscripts I and II refer to the first and second degradation stages, respectively. R: residue at 700 ºC. ζ zeta potential at 25 ºC.
